# Pushing occupational rehabilitation – implementation of a therapy diary in the outpatient aftercare of psychosomatic rehabilitation may promote the occupational reintegration process: a survey of therapists and patients

**DOI:** 10.1186/s12995-021-00306-2

**Published:** 2021-04-21

**Authors:** Carolin Thiel, Cynthia Richter, Franziska-Antonia Zora Samos, Marcus Heise, Thomas Frese, Thomas Fankhaenel

**Affiliations:** 1 Institute of General Practice and Family Medicine, Martin-Luther-University Halle, Magdeburger Straße 8, 06112 Halle (Saale), Germany; 2 SRH Hochschule für Gesundheit GmbH, University of Applied Health Sciences, Campus Gera, Neue Straße 28-30, Gera, Germany

**Keywords:** Psychosomatic rehabilitation, Mental and behavioral disorders, Therapy diary, Outpatient aftercare, Occupational rehabilitation

## Abstract

**Background:**

Treatment results achieved after fulfilling an inpatient psychosomatic rehabilitation are often not permanent. Additional participation in outpatient rehabilitation aftercare may reduce the risk of recurrent disorders and support a successful reentry to working life. A therapy diary should accompany the aftercare and bring about the self-reflection process of psychosomatic rehabilitates, which could reduce recurrent disease progressions and support the recovery process as a whole. The study focuses on the evaluation of the effectiveness and implementation potentialities of a therapy diary in outpatient rehabilitation aftercare.

**Methods:**

In a qualitative study, seven therapists for outpatient rehabilitation aftercare in Central Germany and eleven outpatient psychosomatic rehabilitation patients were interrogated using partially standardized, guideline-based expert interviews.

The data evaluation is based on the Qualitative Content Analysis according to Mayring.

**Results:**

The results show that an accompanying use of a therapy diary during the outpatient rehabilitation aftercare enables an intense commitment through own thoughts and feelings. By writing down thoughts, emotions, dysfunctional behaviors in problematic situations, great successes are experienced. Through this initiated self-reflection process, the rehabilitant gains a better knowledge of one’s behavior in dealing with oneself and the environment and thereby, whenever necessary, learns to create new ways of acting.

**Conclusions:**

The voluntary use of the therapy diary in the outpatient rehabilitation aftercare could assist the therapy process and henceforward the recovery of the rehabilitants, and also increase the prospect of successful occupational rehabilitation.

## Background

According to the Federal Health Monitoring System, the granting of reduced earning capacity pensions, about a third of which can be associated with psychiatric disorders, has almost doubled in the last decade [1, 2]. When it comes to treating psychosomatic disorders, psychosomatic rehabilitation holds the second largest percentage of all rehabilitation treatments [3]. As research results reveal, targeted treatment outcomes after finishing an inpatient psychosomatic rehabilitation are often not consistent [4]. Following the inpatient psychosomatic rehabilitation treatment, the outpatient rehabilitation aftercare should train the patients to cope with their allday life and potentially reoccurring symptoms [5, 6]. Within outpatient rehabilitation aftercare, the affected patients do indeed benefit from interaction and social support during group sessions, both with and through other group members. Hereabouts, such aspects as their health condition and psychosocial obstacle situations are addressed and can then actively be processed in role plays, etc. The exchange also contributes a feeling of shared identity with other patients concerned [7, 8]. Besides, cognitive intervention approaches are used in the context of rehabilitation aftercare. In a series of studies and meta-analyses, used both in individual and group settings, its effectiveness has indeed been proven [4, 9, 10]. The process of self-reflection is one method. By recording their individual experiences, patients are assisted in gaining clarity about their thoughts, emotions, and behaviors. This furthermore, and as a result, serves to identify and treat dysfunctional behavior patterns [11]. The aim of using this reflection process alongside aftercare is to increase the amount of commonly performed rehabilitative aftercare services, reduce recurrent disease processes, and mounting consequential costs. Furthermore, the reflection process allows bolstering the recovery process, including reintegration into working life. Based on the described concept of self-reflection and in the context of the study, an intervention strategy in form of a therapy diary was designed and was used as a means of support within outpatient rehabilitation aftercare groups. The experiences of a) therapists working in outpatient rehabilitation aftercare, and b) outpatient psychosomatic rehabilitation patients that actively use the therapy diary were compiled to answer the following questions: 1) How do you rate the effectiveness of a therapy diary? 2) How can the therapy diary be efficiently implemented in the outpatient rehabilitation aftercare setting?

## Methods

### Study design

The presented study was conducted as side research of a randomized intervention survey. Information on sample and method can be found separately.^1^ For the side study, a qualitative research design was chosen and implemented in the form of partially standardized guideline-supported expert interviews (*N* = 18).

### Sampling

To form the expert group “therapists”, a full survey of all aftercare facilities in Central Germany (Thuringia, Saxony, Saxony-Anhalt) was sought. A total of 37 therapists for outpatient rehabilitation aftercare received a mailing call to participate in the study. Eventually, seven therapists (*n* = 7) for outpatient rehabilitation aftercare were able to take part in the investigation. All of them had at least two years’ professional experience in the outpatient rehabilitation environment.

To compose the expert group “rehabilitation patients” for the study, a total of 81 outpatient psychosomatic rehabilitation patients were recruited to participate. Merely eleven outpatient psychosomatic rehabilitation patients (*n* = 11) took part in the study. All of them actively attended outpatient rehabilitation aftercare and used the therapy diary. Table 1 summarizes the therapist characteristics and Table 2 reviews those of the outpatient psychosomatic rehabilitation patients.

**Table 1:** Characteristics of interviewed therapists (*n* = 7) in absolute frequencies (and percent)

Characteristics of therapists (n = 7)		Frequency of entries n (%)
**Sex**	Female	5 (71)
	Male	2 (29)
**State**	Thuringia	4 (57)
	Saxony	2 (29)
	Saxony-Anhalt	1 (14)
**Professional qualifications**	Psychologist	4 (58)
	Psychotherapist	1 (14)
	Social therapist	1 (14)
	Chief physician rehabilitation	1 (14)

**Table 2:** Characteristics of the interviewed patients (n = 11) in absolute frequencies (and percent)

Characteristics of patients (***n*** = 11)		Frequency of entries n (%)
**Sex**	Female	9 (82)
	Male	2 (18)
**Status of employment**	Full time employed	5 (46)
	Part-time employment	3 (27)
	Job seeking	3 (27)
**Currently unable to work**	No	9 (82)
	Yes	2 (18)
Diagnosed disease*	Depression	9 (82)
	Anxiety disorder	2 (18)
	Burn-out	1 (9)
	Adjustment disorder	1 (9)
	Anorexia Nervosa	1 (9)
	Chronical Pain	1 (9)
**End of outpatient aftercare**	ended regularly	6 (55)
	canceled prematurely	3 (27)
	still running	2 (18)

* The total data is more than 100%, since multiple answers were possible. The diagnosed disease “Burn-out” is not basically a mental disorder. It is a Z-diagnosis. Nevertheless, Burn-out was added to the list of diagnosed diseases because it combines individual psychological symptom levels

### Recruitment

Therapists were selected by direct request in the facility, whilst rehabilitation patients were recruited via the therapists. Therefore, the rehabilitation patients received study information from the therapists that described the research project. Then, the test subjects who were interested in taking part in the study were able to register themselves with the research team.

### Therapy diary (object of investigation)

**Fig. 1:**
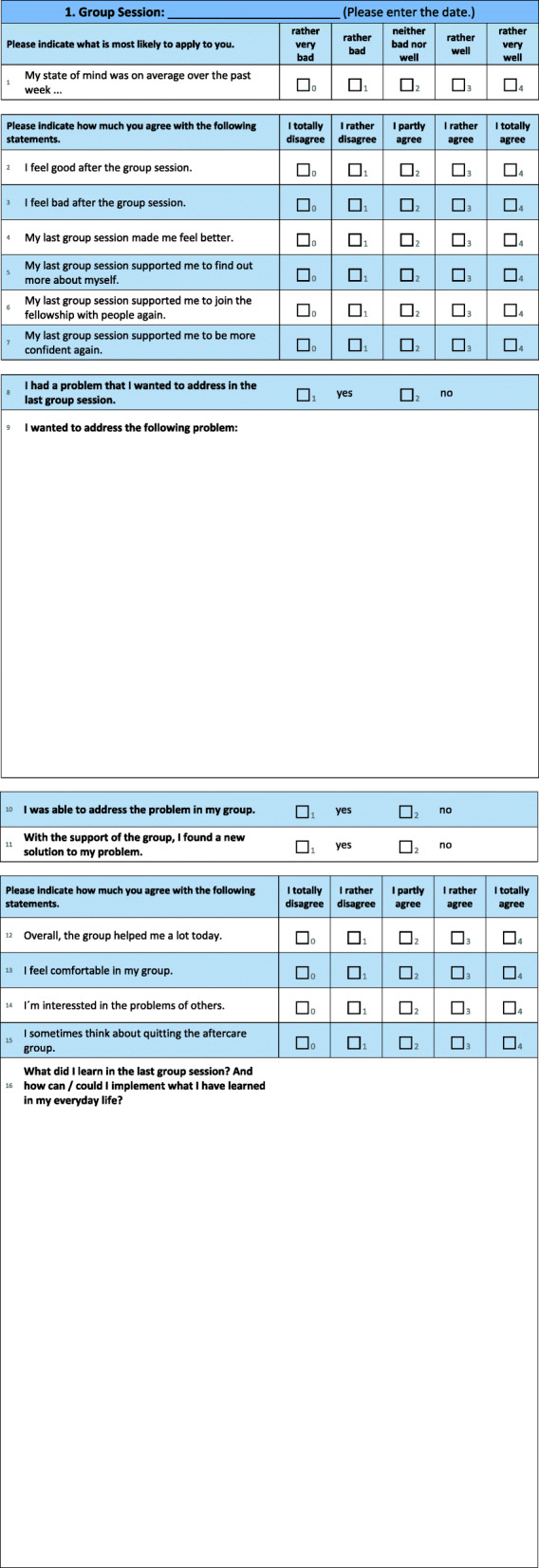
Excerpt from the therapy diary

The focus of this survey was the assessment of a therapy diary that was meant to assist outpatient rehabilitation groups. The therapy diary, which was designed by the research team, consists of a total of 60 pages, set out for 26 corresponding treatment weeks (one session is recorded on a double page, see Fig. 1). The rehabilitation patient answers the same questions every week in closed and open answer setups. Amongst other things, the rehabilitation patient’s overall wellbeing and the groups’ assistance are asked about. The patient has the opportunity to say whether he or she can address individual obstacles within the group. If so, he or she can evaluate which problems they are and whether he or she is supported by the group in finding a solution. To define and pronounce what they have learned from the last session, the rehabilitation patient was also asked to assert in a free text, how he or she was able to implement what he or she has learned in an everyday environment.

### Interview guide (intervention tool)

The basis for the expert audiences with therapists and rehabilitation patients was an interview guide tailored to the test subjects, which served as a framework aid to focus on the individual experiences of the two groups. To answer the research questions, both groups were asked to estimate the effect and impact of motivation to participate in outpatient aftercare and also to reflect the options for implementation of the therapy diary in the therapeutic setting. For this, they were challenged to give their opinion on how the effectiveness of a therapy diary used for outpatient rehabilitation aftercare should be rated; how they judge the intervention diary (in terms of structure, scope, content, clarity, handling, structure, effort, benefit, motivation, etc.); which possible opportunities and difficulties they see through the use of the intervention diary in the outpatient rehabilitation aftercare regarding the quality of the outpatient rehabilitation aftercare, the compliance of the participants and the effect on the dropout rate (reduction through self-reflection by intervention diary use); and nonetheless, how the therapy diary can be efficiently implemented in the outpatient rehabilitation aftercare environment.

### Qualitative analysis

Based on Qualitative Content Analysis according to Mayring [12], the interviews were recorded, transcribed, and assessed. Firstly, a coding system was created, which contained categories defined and based on the interview guideline. A complete revision of the material was then carried out, where corresponding text segments from the individual audiences were assigned to categories. During the explication, the coding system was equipped with additional significant categories. The relevant text elements were then summarized and assessed regarding the underlying topics.

## Results

**1) Assessment of the effectiveness of the therapy diary in the treatment process in outpatient rehabilitation aftercare.**

### Assessment of the effectiveness of the therapy diary

a) Perspective of the therapists:

Overall, the therapy diary was appraised by the surveyed therapists as a tool that supported the treatment process, which can also positively affect the recovery process. The open questions encourage the rehabilitation patients to state what they have learned from previous meetings. Furthermore, this allows reflecting self-critically on whether and in what way this can or has been transferred to everyday life. This way, potential difficulties or concerns can be written down and picked up on directly in the following session, as required, to find a solution with the help of the other group members. Patients who are outgoing and have already particularly ‘reflected’ could benefit from this method.

*“Yes, I also think that it can be beneficial for the participants to see progress more clearly and above all to reflect on it, to overthink what was said and done in the group. And possibly also better prepare for the group sessions when it comes to addressing issues. [ … ] The size is so beneficial. If it’s bigger, it’s going to be harder to always take it with you or have it by hand.” (quote from therapist 01).*

b) Perspective of the rehabilitation patients:

Rehabilitation patients who have regularly completed, or are expected to finish aftercare, evaluated the subjective benefit of participation in aftercare as overall (very) high. The aftercare met expectations and encouraged the pursuit of individual goals in everyday life conditions.

*“It’s really what I wanted. Help for me, tools in hand and a wide range of things you can do to stop falling into deep holes.” (quote from rehabilitation patient 08).*

When asked about the effect of the therapy diary, some of the rehabilitation patients answered that they found the use of the therapy diary helpful when reflecting on and reworking the sessions. Also, when preparing and formulating their discussion requests and goals. However, these were patients who kept their own ‘aftercare diary’ independently of the therapy diary.

*“But I thought it was good to put it all in writing. Yes, it made me stronger, so I truly liked it a lot. To express your feelings once in writing, to search for the words for how I would say it. I still have it all here, like I said, I look at it from time to time.” (quote from rehabilitation patient 07).*

### Suggestions for optimization of the therapy diary design

a) Perspective of the therapists:

As a suggestion for optimization, the introduction of a ‘sub-goals’ page at the beginning, in the middle, and at the end of the therapy diary was recommended. Giving the possibility to compare achieved and previously set goals allows enhancing the reflection process. However, this should not solely be done by the patient, but rather in a two-way dialogue with the therapist. Nevertheless, this may lead to a higher expanse of the session and the appropriate reimbursement.

*“So if there are additional individual sessions, then it should be clear that they will be financed. For sure, an intermediary session would be very useful for some. [...] Well, it would have to be something where it's obvious that if there's a necessity, we can do it. It doesn't have to be regularly, but if things get difficult, it is just inevitable.” (quote from therapist 04).*

b) Perspective of the rehabilitation patients:

In general, an additional intermediate discussion with the therapist in a one-to-one setting is also something that rehabilitation patients increasingly want. This should take place after about half of the appointments in order to reflect on previous progression and the patient’s own development and therefore enable them to enter the second phase of aftercare in a more conscious and targeted manner. The scheme of this intermediate discussion could be based on the records in the therapy diary.

*“Perhaps it would be useful, in addition to the introductory and final discussions, to have the chance to achieve an intermediate step together with the group therapists in a practical manner, one-to-one, to evaluate in that session what the expectations have been and which expectations I do have in real terms.” (quote from rehabilitation patient 01).*

### Additional benefit of implementing the therapy diary in outpatient rehabilitation aftercare

a) Perspective of the therapists:

In the therapists’ opinion, the therapy diary could improve the overall quality of outpatient rehabilitation aftercare as it is a tool that the patient can apply to assist in encouraging him or her even when uncertainties arise in everyday life. In particular and as required, the patient could read back through what progress he or she has made since the beginning of the aftercare. Successfully coping strategies were recorded “in black and white” (quote from therapist 01), which could then be reexamined at a later similar time and applied again in a similar situation.

*“If they come to such an intermediate conclusion, I think that’s OK, then it must be in there: which problems have I implemented or addressed so far? [ … ] which changes have occurred and how have I approached them?” (quote from therapist 02).*

b) Perspective of the rehabilitation patients:

For the participants, one repeatedly mentioned motivation to participate in the aftercare was the desire for therapeutic support after the end of rehabilitation. Aftercare is intended to stabilize the achieved state of health or to prevent a recurrence. To avoid shifting back into old lifestyle and behavior patterns, newly acquired strategies should be strengthened and implemented in everyday life situations. We are aware that the required therapy is not concluded with the end of rehabilitation, but furthermore accompanies the process of reintegration into working life. The rehabilitation patients are willing to follow their personal therapy goals and want to “stick with them”. To underpin this, the therapy diary could be a perfect tool.

*“And above all, [...] so that you just stick with it and do not simply go back home to the everyday's life rat race neglecting everything. Not only making it last 4 to 6 weeks, and then you switch back to your old habits, but you should always remember your personal goals.” (quote from rehabilitation patient 02).*

2) Implementation of the therapy diary in the outpatient rehabilitation aftercare setting.

### Possibilities of implementing the therapy diary in outpatient rehabilitation aftercare

a) Perspective of the therapists:

An evaluation in or with the group would likewise be useful if the outpatient rehabilitation aftercare is taking place in a closed group environment. To support the self-reflection process, the rehabilitation patient needs to receive “external” feedback. Hereabouts, the social support factor from the group would once again come into play, increasing the commitment of the rehabilitation patients. By regularly reporting the partial successes both through the feedback from the group and the therapist, the drop-out rate could be reduced as a result.

*“There it is, model learning. Is someone else’s dilemma interesting, or more even, does it reflect my problem? This means that the patient furtherly recognizes that he or she is not alone with that problem, there are many more people struggling. And if you approve, that serves as an icebreaker. Oh well, I am not alone in this, I may as well let it out. That is also a taboo, isn’t it?” (quote from therapist 02)*

b) Perspective of the rehabilitation patients:

For many of the interviewed rehabilitation patients, it was essential to exchange experiences with other group members, for instance, on topics that have been recorded in the diary. It is also seen as a chance, to being able to learn from the companions’ experiences and to receive positive or even critical feedback, specific tips, and advice regarding their issues. In dialogue with other group members, the rehabilitation patients felt understood, accepted, and bolstered, and in some cases even experienced relativization of their predicaments.

*“It is like being in any crisis area. If you see suffering somewhere else, you will start to perceive some earlier observations quite differently, although you might have seen things more narrowly earlier. [ … ] I have realized it could be worse.” (quote from rehabilitation patient 03).*

### Use of the therapy diary in the outpatient rehabilitation aftercare setting

a) Perspective of the therapists:

Regarding the usage possibilities of the therapy diary within outpatient rehabilitation aftercare, the interviewed therapists highlighted that it was simple to work with, and that the clarity and range of the questions were good. The rehabilitation patients also reported that they valued the use of the diary as overall straightforward and considered the questions to be clearly and easily structured. The therapists, however, stressed the voluntary character of the diary and the absence of penalties for non-use as extremely important. The intervention diary should therefore not be an obligatory part of the aftercare, as there are rehabilitation patients who are less open to this method or who have limited access to it. Some rehabilitation patients may feel overburdened by “schooling” and, as a result, their motivation to participate could be jeopardized.

*“It is the kind of suggestion you get, and I would be in favor of it if it is voluntary. Of course, if you desire to do it, you can do it. But if you do not want to, you do not have to. Then, I think it can be a good thing.” (quote from therapist 03).*

The therapists consider the use of the therapy diary at the beginning of the respective sessions as very beneficial. It enables an immediate start with the rehabilitation patients’ relevant or urgent topics and, if required, to identify common topics and to prioritize them accordingly. Nonetheless, this requires the motivation of the participants.

*“Well, I would first try to use the diary actively, so that the participants bring it into the group settings where they use it as a basis for what they say in the initial discussion round. Then they can work from there. Of course, this only makes sense if the majority of the participants actually do it. And if it cannot be done that way, if I had it available, I would at least offer it.” (quote from therapist 01).*

b) Perspective of the rehabilitation patients:

The rehabilitation patients highlighted furthermore, that managing the therapy diary routinely can be an additional burden if there is too little time for it in everyday life.

*“So the very first few times, you sit down straight after the therapy session and do it right away, that’s OK. But if you come home at 8:30 pm, you do not have enough energy to do it. And when would you do it then? Maybe at the weekend. And then you will have to go back through it all, and if I’m quite honest, you actually do not want to.” (quote from rehabilitation patient 06).*

From the rehabilitation patients’ point of view, using the therapy diary as an introduction to the current session is also considered to be very beneficial. Patients can write notes in advance of what they would like to address in the next session, a question, or a concern that may have remained outstanding since the last sitting. The notes from the previous session should be written as soon as possible. This way, it requires way less effort.

*“If I did not write it straight away in the evening, I would do it first thing the next morning because then I still had everything that was discussed in my mind. So it was no trouble for me at all. You can write it down in no time.” (quote from rehabilitation patient 05).*

## Discussion

The center of this qualitative research, to answer the following questions, has been the experience from outpatient psychosomatic rehabilitation patients, as well as from therapists working in outpatient rehabilitation aftercare: 1) How do you rate the effectiveness of a therapy diary? 2) How can the therapy diary be efficiently implemented in the outpatient rehabilitation aftercare environment? To answer these questions, we drew upon the expertise of 7 therapists for outpatient rehabilitation aftercare and 11 outpatient rehabilitation patients.

### Evaluation of the effectiveness of the therapy diary

The therapists considered the therapy diary intervention tool to be suitable for practical use, not only because it was simple to manage and very clear, but also because it widely helped the therapy process. By writing down thoughts, emotions, and dysfunctional behavior in difficult situations but also the experienced triumphs, the therapy diary allows the patient to scrutinize his or her thoughts and feelings. This initiated self-reflection process enables the patient to gain a better perception of his or her behavior while dealing with himself or herself and the environment. Besides, it permits the patient to reflect and, wherever necessary, to create innovative ways of acting [11]. This way, already achieved therapy goals during the inpatient setting could be strengthened on a long-term basis and will then serve to support the reintegration process into working life. This is expressly the case if the therapists’ approach for optimization of the diary is followed and additional pages are inserted. These can be added at the beginning, halfway through, and at the end of the aftercare to write down “(sub-)goals” which could then serve as a reporting sheet. These could then also be picked up in the two-way dialogue where the patient has the opportunity to receive feedback from the therapist. In the context of therapy, regular feedback is of enormous significance. The patient is given feedback by his or her environment on his or her individual development in course of the treatment process. That enables him or her to create a real self-image: by comparing self-perception with the perception of others [13, 14]. Besides, an evaluation in or with the group would also be useful to support the self-reflection process. This is because current challenges of everyday life and possible solutions are discussed in a group setting during rehabilitation aftercare, which can also especially strengthen self-competence. Self-competence is considered a valuable resource in the context of social participation [15] and can strengthen the process of reintegration into working life. Through direct feedback in or by the group, the rehabilitation patient experiences additional support from the other patients [7, 8], which can then positively influence emotional strengthening. It is indeed a kind of identification of similar experiences with other affected persons [16]. Mostly, this is something that relatives or the individual environment can only do in a limited range, but which is an important factor for therapy motivation and the recovery process. Social support can increase the willingness to participate in therapy [17]. All in all, outpatient rehabilitation aftercare is not only aimed at eliminating chronic (e.g. generalized anxiety disorders, personality disorders) or recurring (e.g. recurrent depression) psychosomatic symptoms, but more specifically to train rehabilitation patients to deal with their disease (and symptoms), which may be dormant or recur for a lifetime. Rehabilitation thus aims to train rehabilitation patients in their coping skills so that they can cope with recurring symptoms [18]. The therapy diary should be seen as a supportive tool that can drive this process forward.

### Possible implementation of the therapy diary in the outpatient rehabilitation aftercare setting

As therapists and rehabilitation patients state, using the therapy diary would be suitable for both: as an introduction to the individual group sessions, as well as a support for the therapy process. It allows rehabilitation patients to think through and to write down their concerns, difficulties, and issues in advance. In the latter case, the therapy diary would serve as documenting progress where accomplishments and failures are recorded and reflected upon. Even more, functional behaviors could be extrapolated, established, and advanced. The therapy diary triggers self-reflection and therefore supports this process. It encourages the patients to better understand their thoughts, emotions, and behavior and enables them to take appropriate action [11]. To implement the therapy diary, the therapists also recommended using it voluntarily. In outpatient rehabilitation aftercare, the therapy diary should therefore be offered and used as a supplement. It should be emphasized from the very beginning that there are no penalties if they do not, or only partially use the therapy diary. Otherwise, the risk raises that some rehabilitation patients would feel overburdened by the additional “effort” of keeping the therapy diary. This resulting in reducing their motivation to participate, and in the worst case leading to discontinuation of aftercare. This was confirmed by the rehabilitation patients. Contrarily, early discontinuation endangers a successful return to working life and also jeopardizes a reduction of the risk of recurrent disorders [5, 19], which are two of the principal goals of outpatient rehabilitation aftercare. In the course of outpatient rehabilitation aftercare and according to the available results, the therapy diary seems to be a suitable supplementary and voluntary option, especially for rehabilitation patients with mental and behavioral disorders. It can sustainably advance the progression of the therapy success by train the patients to cope with their allday life and potentially reoccurring symptoms as well as the reintegration into working life.

#### Strengths and limitations

In this study, therapists in outpatient rehabilitation aftercare with previously tested knowledge in care practice were interviewed. That experience suited practical application and the additional prerequisites of formal training. They also had a wealth of experience with psychosomatic rehabilitation patients who actively worked with the therapy diary. This made it possible to obtain the expertise of proven and effective aftercare experiences that support the recovery process. Furthermore, it allowed achieving a substantiated evaluation of the effectiveness of the therapy diary as well as possible implications. As a matter of fact, due to the number of participants (18), this qualitative study is not a representative sample. Nevertheless, the criterion of theoretical saturation was clearly met. It must also be disclosed that the interviews were particularly attended by rehabilitation patients with an overall motivation to keep a therapy diary. Among these patients, the acquiescence was generally high, which meant that they were more focused on the benefits of the therapy diary. In a further analysis and to determine (in) congruent results, it would be useful to compare the results of the therapist’s and rehabilitation patients’ interviews with the experiences of other workers in the healthcare system who operate in the structure of outpatient rehabilitation aftercare groups. This could create a possibility to define further differentiated implementation options and applying recommendations, which then methodically lead to generalizable results in a quantitative study.

## Conclusion

Occupational rehabilitation is not only focusing on symptom reduction. Occupational rehabilitation as well means training, coping and activity and can also be done despite of recurring or lasting symptoms. Also the therapy diary is focusing on what patients have learned, which is also a coping-orientation rather than a symptom-orientation. For rehabilitation patients, the voluntary use of the therapy diary in outpatient rehabilitation aftercare could bolster the course of the therapy, the recovery, and also the process of reintegration into working life. Especially if the notes serve as a type of documentation of the therapy advancement: for practical behavior tips and coping tactics which result in direct feedback. This feedback could take place in a two-way dialogue with the therapist, but fundamentally also in a group setting. Purposely, the additional factor of social support from the group could increase the patients’ commitment. By regularly recording the respective partial victories during the course of the therapy, the already achieved progress could be advanced. Besides, the chances of successful reintegration into working life could also be increased by train the patients to cope with their allday life and potentially reoccurring symptoms as well. The topic, whether patients who use the therapy diary have more sustainable outcomes than patients who do not use a therapy diary, should be examined in a forthcoming analysis.

## Data Availability

Due to German national data protection regulations, the dataset of the current study is not publicly available but is available from the corresponding author on reasonable request.

## Footnotes

1 *Samos F-A, Thiel C, Richter C, Heise M, Frese T, Fankhaenel T. Outpatient aftercare services can reduce physical and psychological symptoms – The effectiveness of a patient diary in improving symptom burden in patients with mental and behavioural problems in outpatient aftercare in Central Germany: a cluster-randomised controlled trial. 2021. Submitted to “BMC Psychology”.*
